# High-solid Anaerobic Co-digestion of Sewage Sludge and Cattle Manure: The Effects of Volatile Solid Ratio and pH

**DOI:** 10.1038/srep35194

**Published:** 2016-10-11

**Authors:** Xiaohu Dai, Yang Chen, Dong Zhang, Jing Yi

**Affiliations:** 1 State Key Laboratory of Pollution Control and Resources Reuse, School of Environmental Science and Engineering, Tongji University, 1239 Siping Road, Shanghai 200092, China

## Abstract

High-solid anaerobic digestion is an attractive solution to the problem of sewage sludge disposal. One method that can be used to enhance the production of volatile fatty acids (VFAs) and the generation of methane from anaerobic digestion involves combining an alkaline pretreatment step with the synergistic effects of sewage sludge and cattle manure co-digestion, which improves the activity of key enzymes and microorganisms in the anaerobic co-digestion system to promote the digestion of organic waste. In this study, we describe an efficient strategy that involves adjusting the volatile solid (VS) ratio (sewage sludge/cattle manure: 3/7) and initial pH (9.0) to improve VFA production and methane generation from the co-digestion of sludge and manure. The experimental results indicate that the maximum VFA production was 98.33 g/kg-TS (total solid) at the optimal conditions. Furthermore, methane generation in a long-term semi-continuously operated reactor (at a VS ratio of 3/7 and pH of 9.0) was greater than 120.0 L/kg-TS.

Activated sludge technology is a widely used biological method for wastewater treatment, particularly municipal wastewater treatment; however, large amounts of sewage sludge, which must be completely disposed of to avoid secondary pollution to the environment, are produced in this process. Anaerobic digestion has been recognized as one of the most technically mature methods for treating various types of organic waste and offers a high degree of stabilization, reduces waste volume and transforms organics into energy biogas (60–70% of methane). In recent years, high-solid anaerobic processes have attracted increased attention owing to the advantages of a smaller reactor volume, lower energy requirements, and less material handling^1^,^2^,^3^. Because more than 80% of the sewage sludge in China has already been dewatered before further disposal or treatment, high-solid anaerobic digestion has become an attractive solution to the problem of sewage sludge disposal, addressing the inadequate treatment provided by traditional low-solid anaerobic digestion processes^4^. Nevertheless, anaerobic digestion of sewage sludge as the sole substrate has some disadvantages, such as low efficiency of organic conversion, long retention time and low yield of methane; during the high-solid anaerobic digestion of dewatered sludge, the high ammonia content becomes an inhibitory factor^5^.

Anaerobic co-digestion has several benefits for the digestion of different materials, including increased cost-efficiency, the synergistic degradation of treated materials, optimal moisture and nutrient concentrations, the dilution of inhibitory compounds such as ammonia, the degradation of products such as lipids, and the production of biogas^6^,^7^. There are numerous studies on the application of anaerobic co-digestion of sewage sludge with other types of organic waste^8^,^9^ (e.g., a mixture of sewage sludge and strong confectionery waste, a mixture of food waste and cattle manure) because this approach enables the use of existing digesters in wastewater treatment plants (WWTPs) to improve the production of biogas. However, mono-anaerobic digestion of cattle manure has been reported to be unstable owing to the low C/N ratio^10^. Furthermore, a high concentration of animal manure with a total solid content of more than 15% and a large concentration of ammonia and organic nitrogen make these substrates difficult to digest alone^11^. Zhang *et al*.^11^ studied the co-digestion of food waste and cattle manure and found that the production of methane in batch experiments was improved by 41.4% with an optimum ratio of 2 (food waste to cattle manure), which produced a yield of methane of 388 mL/g-VS (volatile solid). Cattle manure was also co-digested with pretreated corn stover, and the results indicated that anaerobic co-digestion obtained a maximum methane yield of 194 mL/g-VS with an optimum corn stover to cattle manure ratio of 3. However, there is little information regarding the use of high-solid anaerobic co-digestion of sewage sludge and cattle manure to improve volatile fatty acid (VFA) production and methane generation.

During the anaerobic digestion process, methane generation from waste solids undergoes three stages: hydrolysis, acidification and methane generation. The hydrolysis of complex organic substances in various wastes is considered the rate-limiting process in the anaerobic digestion system^12^. Because hydrolysis is the rate-limiting stage of methane production, most studies have focused on accelerating sludge hydrolysis using thermal, thermal-alkaline, ultrasonic, mechanical, or thermo-chemical pretreatment strategies. An important strategy to enhance the bioconversion of organic waste to VFAs is to decrease or prevent the activity of methanogens by controlling the pH under acidic or alkaline conditions^13^. Thus, the pH value is one of the most important operational parameters to improve the rate of hydrolysis and maximize the production of VFAs during anaerobic digestion^14^. Yuan *et al*.^13^ found that sludge hydrolysis and VFA accumulation were significantly enhanced at pH 10 for 8 days, whereas there was little methane generated. These results suggest that to enhance the generation of methane during the anaerobic digestion of organic waste, a two-step anaerobic configuration should be considered. The first stage for efficient hydrolysis and VFA accumulation would involve the pretreatment of co-substrates under the optimum fermentation conditions (including volatile solid ratio and pH). Then, methane is generated from the pretreated sludge at a nearly neutral pH, at which the methanogens show maximum activity^15^,^16^. One method that can be used to enhance the production of VFAs and the generation of methane from an anaerobic digestion system is to combine the alkaline pretreatment step with the synergistic effects of sewage sludge and cattle manure co-digestion, which improves the activity of key enzymes and microorganisms in the anaerobic co-digestion system to promote the digestion of organic waste.

Anaerobic digestion is a complex biological process involving large amounts of bacterial and archaeal populations to carry out the hydrolysis, acidification, and methanogenesis of organic waste. Therefore, it is crucial to comprehensively understand the microbial mechanisms necessary to fundamentally improve anaerobic digestion^7^. Recently, the development of next-generation high-throughput sequencing technologies such as 454-pyrosequencing has enabled us to determine a larger number of sequences in a shorter analytical time. 454-Pyrosequencing has been employed to fully explore the microbial communities in various environments^17^ and has been applied to elucidate the microbial diversity of anaerobic digestion^18^. However, few studies have investigated the use of anaerobic co-digestion of sewage sludge and cattle manure using alkaline pretreatment.

The objectives of this study were^1^ to study the feasibility of using high-solid anaerobic co-digestion of sewage sludge and cattle manure under mesophilic conditions^2^; to investigate the combined effects of the sewage sludge to cattle manure ratio and the initial pH on the production of VFAs and the generation of methane and to determine the optimal conditions and related mechanisms for maximum VFA production and methane generation; and^3^ to analyze the microbial community under the optimal conditions for VFA production and methane generation using 454 high-throughput sequencing in a semi-continuous fermentation experiment.

## Material and Methods

### Sewage sludge and cattle manure

The dewatered sewage sludge sample used in this study was obtained from the Anting WWTP in Shanghai, China. The total solid (TS) content of the raw dewatered sludge was 25.0% (w/w), and the volatile solid (VS) content accounted for 47.0% of the TS. During the experimental process, the dewatered sewage sludge was diluted to 16.0% TS. The cattle manure was collected from a farm in Fengxian in Shanghai, of which the TS was 17.54% (w/w) and the VS was 76.0% of the TS. Both the collected dewatered sewage sludge and the cattle manure were stored at 4 °C after sufficiently mixing for 30 min. The mesophilic seed sludge (as inoculum) was collected from an anaerobic digester from Bailonggang WWTP in Shanghai, China, and the TS was 2.3% (w/w) and the VS was 56.5% of TS. The main characteristics of the substrates and inocula used for the investigations are listed in Table 1.

### Experiment on the effects of the VS ratio on the total production of VFAs and their composition

Batch fermentation experiments to investigate the effect of the VS ratio (sewage sludge/cattle manure) on the total production of VFAs and their composition were conducted in eleven identical 1.0 L anaerobic reactors with a liquid working volume of 0.8 L each. The total VS of each reactor was kept at the same amount (40 g/L). Reactors 1–2 were set as the blank tests: one filled with only sewage sludge, and the other filled with only cattle manure. The other 9 reactors were conducted over a range of nine VS ratios (1/9, 2/8, 3/7, 4/6, 5/5, 6/4, 7/3, 8/2 and 9/1). These 11 reactors were sealed with rubber stoppers and then fermented for 18 days. During the experiments, all the reactors were mechanically stirred at 120 rpm (rotations per minute) and maintained under mesophilic temperature conditions (35 ± 1 °C). Every two days, the accumulated concentration of VFAs and their composition were detected until the concentration was stable. All the following experiments were duplicated, and a one-way analysis of variance (ANOVA) at a significance level of 0.05 was used to analyze the data. Unless otherwise stated, all reactors were maintained at 35 ± 1 °C.

### Experiment on the effects of different pHs on the production and composition of VFAs, the consumption of the main organic compounds and the concentration of soluble heavy metals

Mechanism batch experiments to investigate the effects of different pHs on the production and composition of VFAs, the consumption of the main organic compounds and the concentration of soluble heavy metals were conducted in ten identical 1.0 L anaerobic reactors with a liquid working volume of 0.8 L each. The VS ratio was maintained at 3/7, and the total VS was 40 g/L. Reactor 1 had no pH adjustment and was set as the blank test, whereas the initial pH of reactors 2–10 were controlled at 4.0, 5.0, 6.0, 7.0, 8.0, 9.0, 10.0, 11.0 and 12.0, respectively, by adding 2.0 M sodium hydroxide (NaOH) or 2.0 M hydrochloric acid (HCl). The other operational conditions were identical to those described above. Every two days, the production and composition of VFAs, the consumption of the main organic compounds (i.e., proteins, celluloses, hemicelluloses and lignins), and the concentrations of soluble heavy metals were detected.

### Synergistic effects of the main organic compounds in the sludge and manure mixture on the consumption of organic compounds and the production of VFAs

Based on the characteristics of the sludge and manure, batch experiments were conducted using BSA (bovine serum albumin), cellulose, xylan and lignin as model proteins, celluloses, hemicelluloses, and lignins, respectively. To evaluate the synergistic effects of the main organic compounds in the sludge and manure mixture on the consumption of organic compounds and the production of VFAs, four identical 1.0 L anaerobic reactors containing the following organic compounds (g-COD/L) were used: 49.65 BSA (Reactor 1, model sludge only), 11.25 BSA + 18.09 cellulose + 9.72 xylan + 8.54 lignin (Reactor 2, model manure only), 22.8 BSA + 12.69 cellulose + 6.88 xylan + 6.13 lignin (Reactor 3, model mixture of sludge and manure), 22.8 BSA + 12.69 cellulose + 6.88 xylan + 6.13 lignin + pH 9 (Reactor 4, model mixture of sludge and manure at pH 9.0). The organic compounds were dissolved in 800 mL of synthetic wastewater containing (mg/L) 405 NaHCO_3_, 155 K_2_HPO_4_·3H_2_O, 50 CaCl_2_, 100 MgCl_2_·6H_2_O, 25 FeCl_2_, 10 NaCl, 5 CoCl_2_·6H_2_O, 5 MnCl_2_·4H_2_O, 2.5 AlCl_3_, 15 (NH_4_)_6_Mo_7_O_24_, 5 H_3_BO_3_, 5 NiCl_2_·6H_2_O, 5 CuCl_2_·5H_2_O, and 5 ZnCl_2_^19^. The other operational conditions were identical to those described above. Every two days, the production of VFAs, the consumption of the main organic compounds (i.e., protein, cellulose, hemicellulose and lignin), and the concentrations of soluble heavy metals were detected.

### Semi-continuously operated reactors for VFA production and methane generation

Six identical reactors were operated semi-continuously to investigate VFA production and methane generation. The reactors were stirred at a rate of 80 rpm with 10 min of stirring and 10 min of rest. The temperature was maintained at 35 ± 1 °C. The semi-continuous fermentation experiments were operated in three sets of two-phase anaerobic digestion systems. Three reactors (R1, R2 and R3) with a working volume of 7.0 L each were used for the VFA-production phase of the anaerobic digestion process and were fed with sewage sludge, cattle manure and a mixture of sludge and manure (at a VS ratio of 3/7 and pH 9.0), respectively. The other three reactors (R4, R5 and R6) with a working volume of 9.0 L each were used for the methane-production phase of the anaerobic digestion process and were fed with the discharge of R1, R2 and R3, respectively. Reactors 4–6 were flushed for 10 min with nitrogen gas (200 mL/min) to ensure anaerobic conditions prior to starting the digestion tests and were fed with the fermented substrates retrieved from reactors 1–3, respectively. The solids retention time (SRT) of reactors 1–3 was 12 days, and the SRT of reactors 4–6 was 20 days; similar SRTs have also been applied by other researchers^20^,^21^,^22^. After operating for approximately 3 months, VFA production and methane generation remained relatively stable, and then the analysis of the microbial community was conducted.

### Analysis methods

The analyses of TS, VS and pH were conducted in accordance with standard methods^23^, and the measurements of carbohydrates and proteins were based on previously described methods^24^. The contents of cellulose, hemicellulose and lignin were determined based on methods described by Zhang *et al*.^25^. The total concentrations of heavy metals in the feeding and digested substrates were determined using plasma-optical spectrometry (Perkin-Elmer, Optima 2100 DV, USA). For the analysis of VFAs, samples were taken from the reactors and centrifuged at 13,000 rpm for 10 min. The supernatant was then passed through a microfiber filter (0.45 m), and the filtrate was collected in a 1.5 mL gas chromatography (GC) vial. Formic acid was added to adjust the pH to approximately 2.0 before VFA was analyzed using GC (Agilent Technologies 6890N, CA, USA) with a flame ionization detector. The volume of the biogas produced was measured by the displacement of water. The biogas was collected using a gas collector and sampled using a 1 mL syringe to analyze the methane content, which was measured using a gas chromatograph (GC) (Agilent Technologies 6890 N, CA, USA) with a thermal conductivity detector equipped with Hayseq Q mesh and Molsieve 5A columns.

## Results and Discussion

### Effects of the VS ratio on the total production of VFAs and their composition

**Figure 1:**
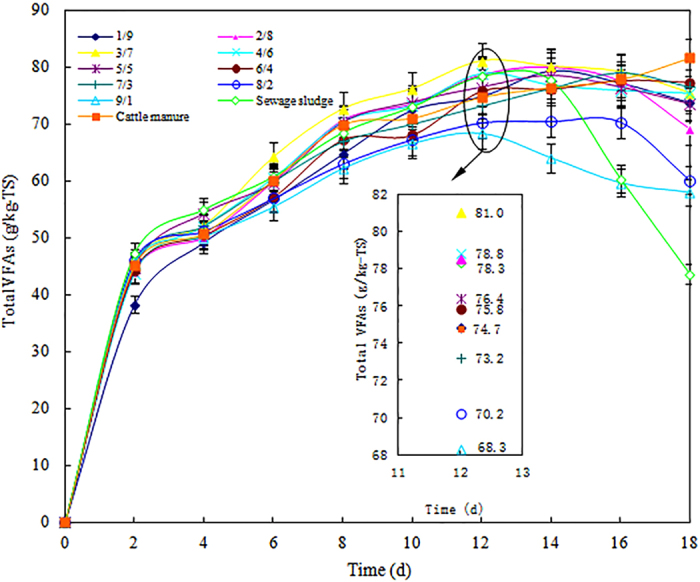
Effects of the different VS ratios on the total production of VFAs during an 18-day fermentation period. The error bars represent the standard deviations of triplicate tests.

The impacts of the different VS ratios on the total VFA production during the 18-day fermentation period are shown in Fig. 1. The results show that the total VFA production at the VS ratio of 3/7 was higher during the 6th to 16th day of fermentation than that at the other VS ratios and the sewage sludge- or cattle manure-only reactors. In the reactor with a VS ratio of 3/7, the total VFA production peaked (81.04 ± 3.65 g/kg-TS) on the 12th day of fermentation, and the total VFA production in the eleven reactors was ordered as follows: VS 3/7 (81.04 ± 3.65) > VS 4/6 (78.78 ± 3.62) > VS 2/8 (78.55 ± 3.38) > sewage sludge only (78.31 ± 3.52) > VS 5/5 (76.41 ± 3.44) > VS 6/4 (75.81 ± 2.43) > VS 1/9 (74.86 ± 2.55) > cattle manure only (74.74 ± 2.47) > VS 7/3 (73.20 ± 2.42) > VS 8/2 (70.23 ± 2.04) > VS 9/1 (68.33 ± 1.71). As shown in Fig. 1, when the VS ratio was 3/7, 4/6, or 9/1 or in the reactor containing only sewage sludge, VFA accumulation peaked on the 12th day, whereas when the VS ratio was 1/9, 2/8, 5/5, 6/4, 7/3 or 8/2, VFA accumulation peaked on the 14th or 16th day. After the peak, VFA accumulation decreased slightly in the nine reactors containing the various VS ratios or only sewage sludge; however, VFA accumulation in the cattle manure-only reactor continued to improve with the fermentation time and reached 81.63 ± 3.84 g/kg-TS before slowing in the latter stages of the digestion process.

The above results showed that the addition of cattle manure enhanced the total VFA production because cattle manure adjusted the VS ratio in the sewage sludge. Although higher VFA production was also achieved using only cattle manure, it took a much longer fermentation time to yield a similar VFA yield that was produced at a VS ratio of 3/7 on day 12 of fermentation. The reason for the reduced VFA production at a VS ratio of 3/7 during the initial 4 days might be that the organic matter in cattle manure is more difficult to digest than the organic matter in sewage sludge. Therefore, the optimal conditions for the production of VFAs are a VS ratio of 3/7 and a fermentation time of 12 days.

**Figure 2:**
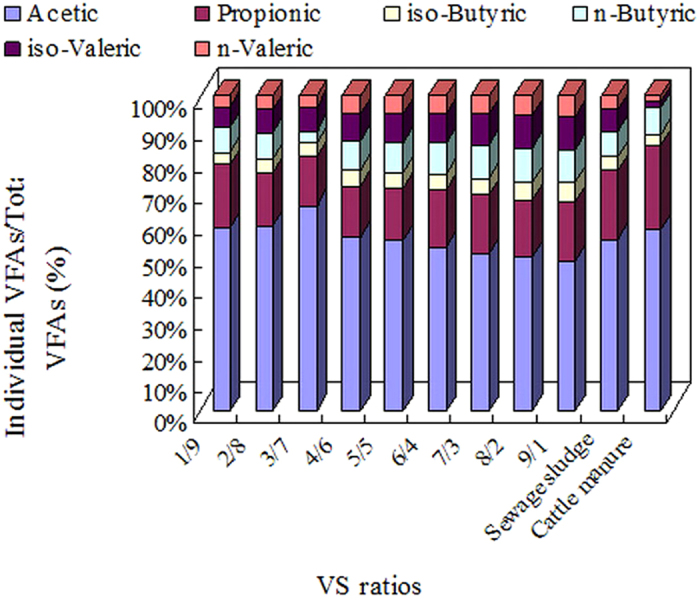
Effects of the different VS ratios on the percentage of individual VFAs accounting for the total VFAs at a fermentation time of 12 days. The data reported are the averages of triplicate tests.

In this study, six VFAs (i.e., acetic acid, propionic acid, iso-butyric acid, n-butyric acid, iso-valeric acid, and n-valeric acid) were observed. The effects of the different VS ratios on the percentage of individual VFAs accounting for the total VFAs at a fermentation time of 12 days are shown in Fig. 2. The results show that acetic acid was the most prevalent product. As the VS ratio increased from 1/9 to 3/7, the percentage of acetic acid accounting for the total VFAs improved from 58.10% to 65.40% but gradually decreased to 47.65% as the VS ratio changed from 3/7 to 9/1. In addition, the percentage of acetic acid in the VS ratio 3/7 fermentation reactor (65.40%) was much higher than in the reactor containing only sewage sludge (54.63%) or cattle manure (57.55%) at fermentation day 12. Furthermore, as shown in Figure S1 (Supplementary Data), the production of acetic acid in the VS ratio 3/7 fermentation reactor was the highest between fermentation days 2 to 18. As shown in Fig. 2, the percentage of propionic acid decreased from 20.16% to 15.25% as the VS ratio changed from 1/3 to 3/7 and then increased as the VS ratio changed from 3/7 to 9/1. For the reactors containing only sewage sludge or only cattle manure, the percentages of propionic acid were 21.70% and 26.87%, respectively. The percentages of the other VFAs were also affected by the different VS ratios. For example, Fig. 2 indicates that the percentage of iso-butyric acid and iso-valeric acid gradually increased as the VS ratio increased from 1/9 to 9/1.

According to Figs 1 and 2, the production of individual VFAs at the different VS ratios can be easily calculated. The maximum yield of acetic acid occurred at a VS ratio of 3/7 (53.00 ± 1.64 g/kg-TS) and a fermentation time of 12 days. Compared with the mixture with a VS ratio of 3/7, the yield of acetic acid in the blank tests (sewage sludge and cattle manure) was only 42.78 ± 1.93 and 43.02 ± 0.82 g/kg-TS, respectively. Acetic acid can be degraded into CH_4_ and CO_2_ directly by methanogens, which should result in low net acetic acid production. Therefore, the acetic acid content in VFAs and the total VFA production could be significantly improved by maintaining the VS ratio at 3/7, which can benefit further utilization in related areas.

### Effects of pH on the production and composition of VFAs from the sludge and manure mixture at a VS ratio of 3/7

**Figure 3:**
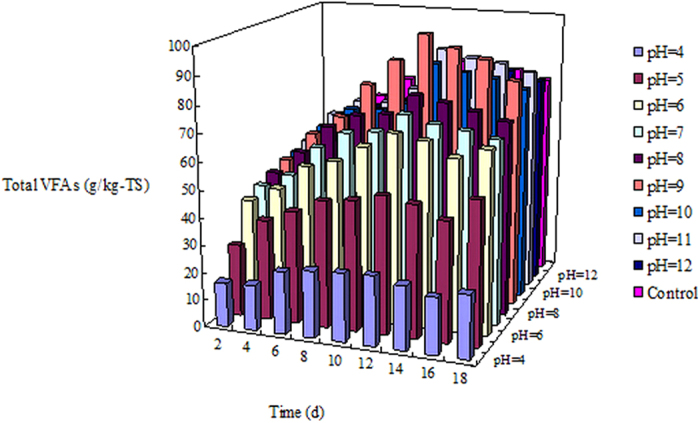
Effects of pH and fermentation time on the total production of VFAs in the sludge and manure mixture at a VS ratio of 3/7. The data reported are the averages of triplicate tests.

The effects of pH and fermentation time on the total production of VFAs in the sewage sludge and cattle manure mixture are shown in Fig. 3. During the initial 6-day fermentation time, the production of total VFAs was as follows: pH 11.0 (66.78 ± 1.87) > pH 10 (65.96 ± 2.44) > pH 9 (65.56 ± 1.10) > blank test (64.36 ± 2.45) > pH 8 (64.05 ± 1.79) > pH 12 (62.74 ± 1.82) > pH 7 (58.82 ± 2.00) > pH 6 (54.78 ± 2.30) > pH 5 (41.12 ± 1.77) > pH 4 (22.82 ± 0.75). As the fermentation time increased to 12 days, the total production of VFAs improved for all cases, and with respect to the production of total VFAs, almost the same order of pH values as was seen for 6 days of fermentation, i.e., pH 9.0 (98.33 ± 4.13) > pH 11 (89.01 ± 3.03) > pH 10 (85.80 ± 3.60) > pH 12 (82.04 ± 3.12) > blank test (80.73 ± 2.91) > pH 8 (78.16 ± 3.05) > pH 7 (73.65 ± 2.06) > pH 6 (69.52 ± 2.36) > pH 5 (50.30 ± 2.26) > pH 4 (25.52 ± 1.15). One significant change was that the highest total VFA production was obtained at pH 9.0. After 12 days, increased fermentation time did not lead to improvements in total VFA production in any of the cases.

The above results showed that the production of VFAs in the sewage sludge and cattle manure mixture could be significantly enhanced by adjusting the pH to 9.0 at the fermentation time of 12 days, which indicates that the pH was different from that of previous studies^13^,^26^. These differences might be due to the different fermentation substrates used in this study, which affected the optimal pH in the VFA-production system. The results suggest that the optimal fermentation conditions for maximum VFA production from a sewage sludge and cattle manure mixture at a VS ratio of 3/7 were a pH 9.0 and a fermentation time of 12 days. The reason for the higher VFA production at pH 9.0 and 12 days will be discussed below. Furthermore, as shown in Fig. 3, obvious VFA consumption was observed in all the pH adjustment tests and the blank test after 12 days of fermentation owing to the participation of VFA consumers such as methanogens.

**Figure 4:**
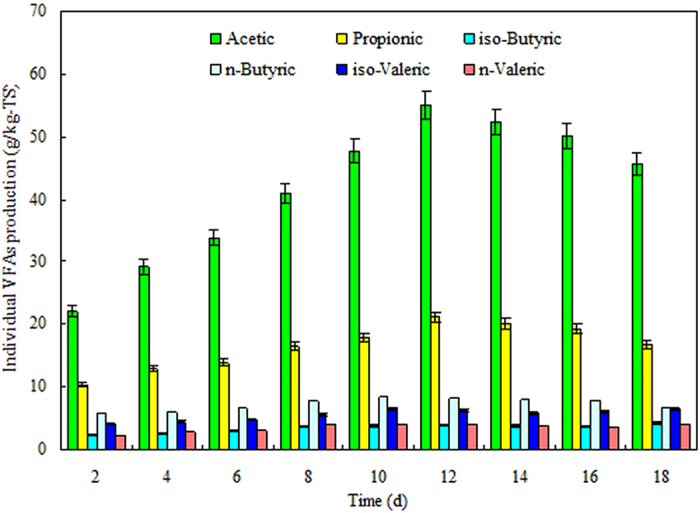
The production of individual VFAs in the sludge and manure mixture at a VS ratio of 3/7 and a pH of 9 during an 18-day fermentation time. The error bars represent the standard deviations of triplicate tests.

The individual VFAs from the sludge and manure mixture at a pH of 9.0 and a VS ratio of 3/7 during the 18-day fermentation time period are shown in Fig. 4. The results show that both acetic acid and propionic acid were the two most prevalent VFAs produced at any of the fermentation times. Acetic acid increased significantly from 22.04 ± 0.51 g/Kg-TS (on the 2nd day) to 55.02 ± 1.87 g/Kg-TS (on the 12th day) and then decreased to 45.75 ± 1.92 g/Kg-TS as the fermentation time increased to day 18. Furthermore, the percentage of acetic acid accounting for the total VFAs ranged from 46.96 ± 1.08% to 55.95 ± 1.90%, which was significantly higher than that of any other VFAs at any of the fermentation times. The other VFAs showed similar trends as acetic acid during the digestion process. Propionic acid was the second most prevalent VFA during the digestion process. Propionic acid also increased as the fermentation time increased from day 2 to day 12, reaching a maximum production of 21.08 ± 0.55 g/kg-TS at a percentage of 21.44 ± 0.56%, before showing a decreasing trend as the fermentation time increased. Furthermore, n-butyric acid and iso-valeric acid accounted for approximately only 5–9% and 4–7% of total VFAs during the fermentation time, respectively. Iso-butyric acid and n-valeric acid were the two least prevalent VFAs produced during the digestion process. Their maximum production was approximately 4.28 ± 0.14 and 4.04 ± 0.17 g/kg-TS, which was approximately 5.11 ± 0.17% and 4.58 ± 0.20% of the total VFAs, respectively.

Because the main aim of this study was to enhance the production of VFAs from sewage sludge by adding cattle manure and adjusting the pH to enhance the methane yield, the mechanisms of higher VFA production and methane generation at a VS ratio of 3/7 and a pH of 9.0 will be discussed below.

### Effects of different pHs on the consumption of the main organics compounds in the sludge and manure mixture

**Figure 5:**
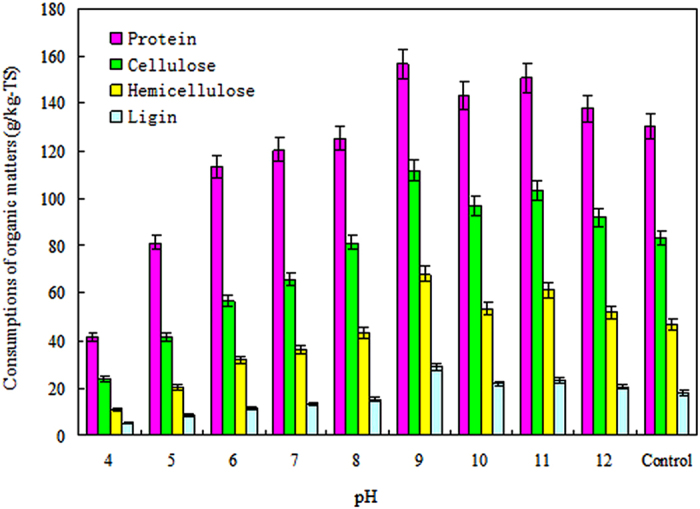
Effects of the different pHs on the consumption of celluloses, hemicelluloses, lignins and proteins at a VS ratio of 3/7 and a fermentation time of 12 days. The error bars represent the standard deviations of triplicate tests.

Because proteins, celluloses, hemicelluloses and lignins were the main constituents in the dewatered sludge and cattle manure mixture (Table 1), we investigated the effects of different pHs on the consumption of the fermentation substrates (proteins, celluloses, hemicelluloses and lignins), which can be bio-converted to VFAs during anaerobic digestion^27^,^28^. The influences of the different pHs on the consumptions of proteins, celluloses, hemicelluloses and lignins in the sewage sludge and cattle manure mixture at a VS ratio of 3/7 and a fermentation time of 12 days are shown in Fig. 5. The results show that the consumption of proteins was higher in the reactors where the pH was adjusted to 4.0–12.0 and the consumption of lignins was lower in the reactors where the pH was uncontrolled. These results suggest that celluloses and hemicelluloses were readily digested and lignins were difficult to break down during anaerobic fermentation. The consumption of these main organic compounds increased as the pH increased from 4.0 to 9.0 but decreased as the pH increased further from 9.0 to 12.0. Furthermore, as shown in Fig. 5, the consumption of proteins, celluloses, hemicelluloses and lignins in the reactor with a pH of 9.0 was higher than in the reactors with the other pHs and the blank test, peaking at 156.55 ± 3.26, 111.83 ± 2.14, 68.00 ± 0.97 and 29.20 ± 0.47 g/kg-TS, respectively. These results are in agreement with the finding that the total concentration of VFAs was the highest at pH 9.0.

### Synergistic effects of the main organic compounds in the sludge and manure mixture on the consumption of organic compounds and the production of VFAs

To further verify the effect of adding cattle manure and adjusting the pH on the total production of VFAs, batch tests using model protein and model carbohydrate compounds (i.e., cellulose, hemicellulose and lignin) were conducted. As shown in Table S1 (Supplementary Data) proteins, celluloses, hemicelluloses and lignins decreased in each reactor at fermentation day 12, which indicated that they were consumed by the microbes in the sewage sludge and cattle manure mixture. In addition, regardless of whether the main substrate was protein, cellulose, hemicellulose or lignin, the total consumption of these substrates in the sludge and manure mixture at pH 9.0 was significantly higher than in the other tests, which was in agreement with the findings that the total VFA yield was the highest in the reactor containing a mixture of sewage sludge and cattle manure at pH 9.0 (Table S2, Supplementary Data). All these results suggest that VFA production from only sewage sludge and cattle manure or the mixture of the two was related to the fermentation of its proteins and carbohydrates, and the co-fermentation of sewage sludge and cattle manure at pH 9.0 increased the production of total VFAs.

### Effects of different pHs on the concentrations of soluble heavy metals in the fermentation reactors

It was previously reported that many heavy metals are part of the essential enzymes that catalyze numerous anaerobic reactions^29^, and the increase in the content of heavy metals improves anaerobic digestion^30^. Therefore, it is necessary to study the impact of pH on the contents of soluble heavy metals in the fermentation reactors. Table 2 shows the concentrations of soluble heavy metals in the fermentation reactors at different pHs on the 12^th^ day of fermentation. The content of Fe^3+^ was the highest of all the heavy metals detected for the different pH adjustments and the blank test. The highest Fe^3+^ release (11.47 ± 0.24 g/kg-TS) occurred at pH 9.0. Our previous research also showed that the highest Fe^3+^ concentration in the fermentation reactor was consistent with the highest VFA and methane production^14^. Therefore, one reason that the pH 9.0 adjustment to the high-solid sewage sludge and cattle manure mixture showed the greatest VFA production was due to the highest Fe^3+^ release.

### VFA production and methane generation in the semi-continuous fermentation reactors

**Figure 6:**
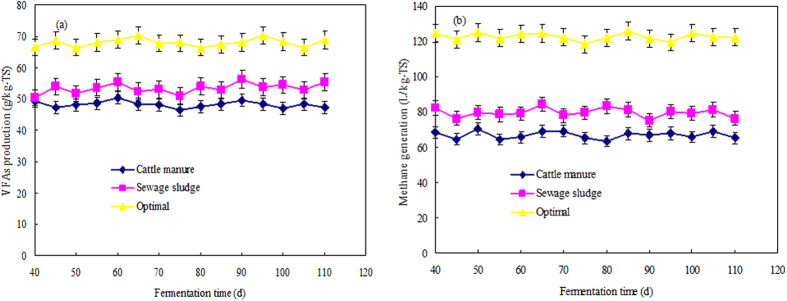
Variations in VFA production (a) and methane generation (b) in the semi-continuous reactors containing only sludge, only manure and the mixture (at pH 9) during a 120-d fermentation period. The VFAs consisted of acetic acid, propionic acid, iso-butyric acid, n-butyric acid, iso-valeric acid, and n-valeric acid. The error bars represent the standard deviations of triplicate measurements.

Figure 6 shows variations in VFA production and methane generation in three long-term semi-continuously operated reactors over a fermentation period of 120 days. The results show that the average VFA production was 67.99 ± 1.44 g/kg-TS in the reactor containing the mixture (at a VS ratio of 3/7 and pH of 9.0), whereas the average VFA production was only 53.36 ± 1.21 g/kg-TS and 48.29 ± 2.09 g/kg-TS in the reactors containing only sludge or manure, respectively. Therefore, even in the long-term experiment, VFA production was still significantly higher in the sewage sludge and cattle manure mixture (at pH 9.0) than in the reactors containing sludge or manure only. Based on the comparison of the data in Figs 1 and 6a, VFA production in the long-term experiment was lower than in the batch tests. Similar observations were found in previous studies. Yuan *et al*.^13^ reported that approximately 250.0 mg COD/g VSS of VFAs was generated in batch experiments, whereas Feng *et al*.^31^ indicated that only 95.0 mg COD/g VSS of VFAs was produced in semi-continuous tests. All the above results clearly show that the use of mixtures of sewage sludge and cattle manure with the pH adjusted to 9.0 enhances both VFA production and accumulation.

VFAs produced in the acidification process will be further bio-converted to methane in the subsequent methanogenesis stage. As shown in Fig. 6b, the methane production, after reaching stability in the digestion reactor with a pH of 9.0 and a VS ratio of 3/7, was observed to be greater than 120.0 L/kg-TS, whereas in the reactors containing sludge or manure only, the methane production was 79.66 ± 2.67 L/kg-TS and 66.86 ± 1.76 L/kg-TS, respectively. The high-solid anaerobic co-digestion of sludge and manure with the pH adjusted to 9 significantly improved the methane production compared to the anaerobic mono-digestion of sludge and manure. Previous studies have indicated that under optimum conditions, more total VFAs with higher levels of acetic acid were produced, more organic matter was consumed, more synergistic effects of the main organic compounds were observed, more soluble heavy metals were released, and greater concentrations of bacteria and archaea were produced. This might be the mechanism by which higher methane is generated at a VS ratio of 3/7 and pH of 9.0.

### Analysis of the microbial community in the anaerobic fermentation system at a VS of 3/7 and pH of 9.0

**Figure 7:**
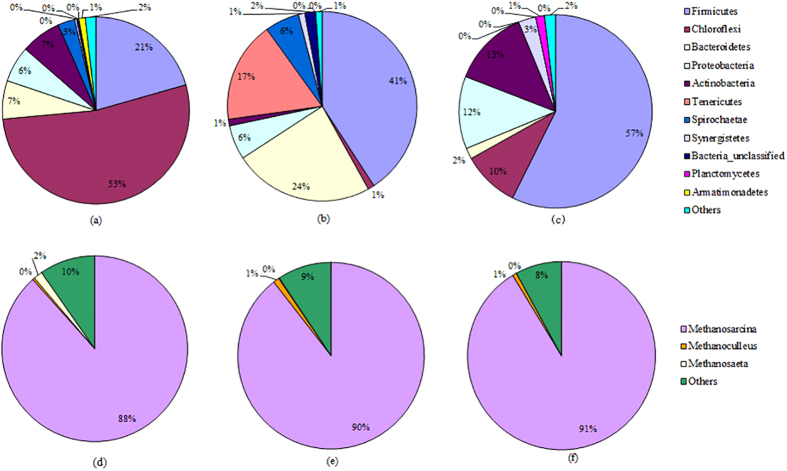
Phylum-level distribution of *Bacterial* populations (a–c) and genus-level distribution of *Archaeal* populations (d–f) in the anaerobic digestion system containing only sludge (a,d), only manure (b,e) and the mixture (c,f).

Anaerobic digestion is a complex process involving large bacterial and archaeal populations to carry out the hydrolysis, acidification and methanogenesis of organic matter, and the microbial characteristic assay has been proven to be a useful tool with which to evaluate the performance of anaerobic systems in terms of VFA production and methane generation. Therefore, we investigated the reason the high-solid co-digestion of sludge and manure resulted in increased VFA and methane production by analyzing the microbial community using 454-high-throughput pyrosequencing. The results are shown in Table S3 and Fig. 7.

Figure 7 (a–c) illustrates the phylum level distributions of bacterial OTUs in the reactors containing only sewage sludge, only cattle manure and the mixture (at pH 9.0). Most of the bacteria in the anaerobic digestion reactors were grouped into *Firmicutes*, *Chloroflexi*, *Bacteroidetes*, *Proteobacteria*, *Actinobacteria*, *Actinobacteria*, *Tenericutes*, *Spirochaetae*, and *Synergistetes*. Similarly, a meta-analysis of results available in public databases showed that 16,519 bacterial sequences were retrieved from conventional anaerobic digesters and that *Firmicutes*, *Chloroflexi*, *Bacteroidetes* and *Proteobacteria* were the main bacterial phyla^32^. However, the results of our experiment showed that *Firmicutes*, *Chloroflexi*, *Bacteroidetes* and *Proteobacteria* accounted for 40.8%, 1.1%, 23.9% and 5.9%, respectively, of total bacterial sequences in the anaerobic reactor containing only sludge, whereas in the reactor containing only manure, they accounted for 20.6%, 52.9%, 6.6% and 6.3%, respectively, and 57.4%, 9.6%, 1.8% and 12.1% in the reactor containing the mixture (at pH 9.0). These results indicate that digestion in the mixture at pH 9.0 improved the relative abundance of *Firmicutes* in the anaerobic digestion reactors.

The VFAs produced in the acidification process will be consumed in the subsequent methanogenesis stage. Figure 7 (d–f) further illustrates the genus level distribution of the archaeal populations in the fermentation reactors. In the reactor containing only sludge, the genera *Methanosarcina*, *Methanoculleus* and *Methanosaeta* accounted for 88.4%, 0.4% and 1.5% of total archaeal sequences, respectively, whereas they accounted for 89.5%, 1.0% and 0.1%, respectively, in the reactor containing only manure. However, the archaeal sequences from the reactor of the mixture (at pH 9.0) was mainly related to *Methanosarcina sp*. (91.3%), whereas the abundance of *Methanoculleus sp*. and *Methanosaeta sp*. decreased to 0.7% and 0.1%, respectively. Both *Methanosarcina sp*. and *Methanosaeta sp*. have been reported to be able to convert VFAs, particularly acetic acid, to methane, and these two microorganisms belong to the domain *Archaea*^14^. Nevertheless, *Methanosarcina sp*. can grow at higher acetate concentrations, whereas lower acetate concentrations increase the predominance of *Methanosaeta sp*.^14^, which might explain why the predominant micro-colonies were *Methanosarcina sp*. in the granules using the mixture at pH 9.0 as a substrate (high acetate concentration).

## Conclusion

The above studies investigated a new strategy to improve VFA production and methane generation by controlling the key parameters in a sewage sludge and cattle manure co-digestion system. The optimal conditions for improved VFA production and methane generation from sewage sludge and cattle manure were a VS ratio of 3/7 and an initial pH of 9.0. Further experiments indicated that at the optimum conditions, more organic matter was consumed, more synergistic effects of the main organic compounds were observed, more soluble heavy metals were released, and greater concentrations of bacteria and archaea were produced.

## Additional Information

**How to cite this article**: Dai, X. *et al*. High-solid Anaerobic Co-digestion of Sewage Sludge and Cattle Manure: The Effects of Volatile Solid Ratio and pH. *Sci. Rep*. **6**, 35194; doi: 10.1038/srep35194 (2016).

## Supplementary Material

Supplementary Information

## Figures and Tables

**Table 1:** Characteristics of the sewage sludge and cattle manure used in the experiments^a^.

	pH	TS (%)	VS (g/Kg-TS)	Total protein (g/Kg-TS)	Cellulose (g/Kg-TS)	Hemicellulose (g/Kg-TS)	Lignin (g/Kg-TS)
Sewage sludge	6.3 ± 0.1	16.02 ± 0.35	470.30 ± 5.64	388.89 ± 5.40	—	—	—
Cattle manure	8.2 ± 0.2	17.54 ± 0.21	758.60 ± 6.09	142.50 ± 3.71	254.31 ± 2.19	136.72 ± 3.28	75.01 ± 2.41

^a^Results are the average and their standard deviation of triplicate measurements.

**Table 2:** Effects of different pHs on the concentrations of soluble heavy metals in the fermentation reactors at a VS ratio of 3/7 and a fermentation time of 12 days^a^.

	Al	Ba	Cr	Cu	Fe	Mn	Ni	Pb	Zn
pH 4	0.493 ± 0.020	0.048 ± 0.002	0.026 ± 0.001	0.057 ± 0.003	5.102 ± 0.163	0.158 ± 0.006	0.246 ± 0.005	0.008 ± 0.001	0.332 ± 0.007
pH 5	0.582 ± 0.011	0.058 ± 0.003	0.048 ± 0.002	0.327 ± 0.007	5.782 ± 0.197	0.173 ± 0.008	0.134 ± 0.003	0.012 ± 0.001	0.544 ± 0.017
pH 6	0.668 ± 0.023	0.079 ± 0.002	0.046 ± 0.001	0.634 ± 0.022	7.284 ± 0.277	0.197 ± 0.009	0.191 ± 0.006	0.010 ± 0.001	0.406 ± 0.008
pH 7	0.672 ± 0.018	0.078 ± 0.003	0.044 ± 0.001	0.639 ± 0.029	8.559 ± 0.325	0.207 ± 0.009	0.045 ± 0.001	0.009 ± 0.001	0.437 ± 0.048
pH 8	0.750 ± 0.018	0.089 ± 0.003	0.043 ± 0.001	0.363 ± 0.015	9.337 ± 0.345	0.226 ± 0.004	0.128 ± 0.003	0.012 ± 0.001	0.482 ± 0.023
pH 9	0.616 ± 0.023	0.128 ± 0.005	0.041 ± 0.002	1.224 ± 0.054	11.472 ± 0.241	0.228 ± 0.005	0.489 ± 0.014	0.012 ± 0.001	0.474 ± 0.011
pH 10	0.607 ± 0.028	0.072 ± 0.003	0.037 ± 0.002	0.522 ± 0.023	10.221 ± 0.281	0.165 ± 0.002	0.223 ± 0.007	0.007 ± 0.001	0.364 ± 0.008
pH 11	0.468 ± 0.007	0.055 ± 0.002	0.029 ± 0.001	1.973 ± 0.068	9.087 ± 0.168	0.147 ± 0.007	0.880 ± 0.032	0.011 ± 0.001	0.339 ± 0.004
pH 12	0.383 ± 0.011	0.049 ± 0.002	0.023 ± 0.001	0.048 ± 0.002	7.548 ± 0.151	0.151 ± 0.004	0.031 ± 0.001	0.007 ± 0.001	0.306 ± 0.006
Control	0.636 ± 0.025	0.109 ± 0.003	0.043 ± 0.002	0.104 ± 0.003	10.718 ± 0.654	0.224 ± 0.004	0.067 ± 0.002	0.013 ± 0.001	0.576 ± 0.016

^a^The unit is g/kg-TS, and the TS refers to the initial TS. The data are the averages and their standard deviations in duplicate tests.
