# Supplementary material for: High-solid Anaerobic Co-digestion of Sewage Sludge and Cattle Manure: The Effects of Volatile Solid Ratio and pH
Source: Sci Rep. 2016 Oct 11;6:35194. doi: 10.1038/srep35194 (PMC5057105; doi:10.1038/srep35194)
Supplement: Supplementary Information [file srep35194-s1.doc]

**Supplementary information**

**High-solid Anaerobic Co-digestion of Sewage Sludge and Cattle Manure: The Effects of Volatile Solid Ratio and pH**

Xiaohu Dai, Yang Chen, Dong Zhang[[1]](#footnote-2)*, Jing Yi

*(State Key Laboratory of Pollution Control and Resources Reuse, School of Environmental Science and Engineering, Tongji University, 1239 Siping Road, Shanghai 200092, China)*

^*^Corresponding author

Dong Zhang Phone: 86-21-55126332 Fax: 86-21-65986313 E-mail: zhangdong_2011@aliyun.com

**Supplementary Material:**  8 pages, 3 tables, 3 figures

**454 high-throughput pyrosequencing method**

In order to detect the microbial community of the anaerobic digestion systems, samples were collected directly from the reactors and the total genomic DNA was extracted from each sample using a PowerSoil DNA Isolation Kit (Mo Bio Laboratories Inc., Carlsbad, CA, USA) according to the manufacturer’s instructions. Three successive DNA extractions of each sample were pooled before PCR (to minimize DNA extraction bias). PCR amplifications of the bacterial and archaeal of 16S rRNA genes segments were amplified from the genomic DNA using the highly conserved universal bacterial and archaeal primer sets as described by Guo et al. (*1*). The primer pairs of 27F (5’-AGAGTTTGATCCTGGCTCAG-3’) and 533R (5’-TTACCGCGGCTGCTGGCAC-3’) were employed for bacteria, while 344F (5’-ACGGGGYGCAGCAGGCGCGA-3’) and 915R (5’-GTGCTCCCCCGCCAATTCCT-3’) for archaea. To achieve the sample multiplexing during pyrosequencing, barcodes were incorporated between the 454 adaptor and forward primer. All PCR reactions for each sample were performed in triplicate (including two negative control reactions) with 2.0 μM of each primer, 0.25 μM dNTPs (Takara), 4 μL of 5 × FastPfu Buffer (TransGen, TransGen Biotech Co., Ltd., Beijing, China), 1 U of FastPfu DNA polymerase (2.5 U/μL, TransGen), and 20 ng of DNA template with a final volume of 20 μL. According to the literature (*1*), the thermal cycling for bacteria consisted of initial denaturation at 98^o^C for 2 min followed by 25 cycles of denaturation at 98^o^C for 10 s, annealing at 55^o^C for 15 s, and extension at 72^o^C for 15 s, with a final extension of 5 min at 72^o^C. The thermal cycling for archaea was similar to that for bacteria except that the annealing temperature was 57^o^C.

The PCR products were sent to Majorbio Bio-Pharm Technology Co., Ltd., (Shanghai, China) for sequencing on the Roche GS FLX 454 pyrosequencing platform. The PCR products of each sample were pooled by volume (to minimize PCR bias), purified with the QIAquick PCR Purification Kit (Qiagen, Valencia, CA), and then quantified using a NanoDrop spectrophotometer (NanoDrop Technologies, Wilmington, DE). Next, the PCR products of different samples were pooled in equimolar concentrations in the final mixture, which was used to construct the PCR amplicon libraries according to the manufacture’s instruction. For each library, more than 10,000 sequence reads were retrieved from bacterial community and more than 5,000 sequence reads were retrieved from archaea community. After the sequencing was completed, all sequence reads were quality checked using the Mothur software. The poor quality reads were removed. The adapters, barcodes and primers in all raw sequences were trimmed (*2*), and the sequences containing ambiguous nucleotides, checking as chimeric artifact or less than 200 bp in length were removed. Subsequently, the remaining unique sequences were aligned against the Silva database using Bayesian approach (*3*). Sequences with similarities of greater than 97% were clustered into one operational taxonomic unit (OTU) using the MOTHUR program (*4*). Community richness and diversity indices (Chao1 estimator, ACE, Shannon and Simpson indices) and rarefaction curves were obtained using the MOTHUR program. Mothur was also used to conduct the rarefaction analysis, calculating Good's coverage, abundance-based coverage estimator (ACE), Chao1 richness estimator and Shannon and Simpson diversity indices. The raw sequences have been deposited in the NCBI short read archive (SRA) database under the accession numbers SRA058043 for bacterial sequences and SRA058572 for archaeal sequences.

**Table S1 Changes of protein, cellulose, hemicellulose and lignin in the batch tests using dextran, BSA, cellulose, xylan or lignin as the substrates (the initial COD values of dextran, BSA, cellulose, xylan and lignin were set respectively close to the conditions of sole sludge, sole manure, mixture of sludge and manure at VS ratio 3/7, mixture of sludge and manure at VS ratio 3/7 and pH 9.0) after 12 d ^a^**.

|  | Consumptions of organic matters | | | | |
| --- | --- | --- | --- | --- | --- |
| Model substrates | Protein | Cellulose | Hemicellulose | Lignin | Total |
| B ^b^ | 29.39 ± 1.47 | - | - | - | 29.39 ± 1.47 |
| B+C+H+L ^c^ | 3.96 ± 0.15 | 6.37 ± 0.26 | 3.42 ± 0.15 | 3.05 ± 0.15 | 16.80 ± 0.71 |
| B+C+H+L ^d^  (VS ratio=3/7) | 11.70 ± 0.47 | 6.59 ± 0.32 | 3.68 ± 0.20 | 2.67 ± 0.13 | 24.64 ± 1.12 |
| B+C+H+L ^e^  (VS ratio=3/7),  pH=9 | 12.62 ± 0.51 | 8.80 ± 0.45 | 4.03 ± 0.16 | 3.46 ± 0.18 | 28.89 ± 1.30 |
| ^a^ The units of protein, cellulose, hemicellulose and lignin are g-COD/L, and the TS refers to the initial TS. The data are the averages and their standard deviations in duplicate tests. B: BSA; D: Dextran; C: Cellulose; H: Hemicellulose; L: Lignin.  ^b^ The initial COD value was set close to the condition of sole sludge.  ^c^ The initial COD value was set close to the condition of sole manure.  ^d^ The initial COD value was set close to the condition of mixture of sludge and manure at VS ratio 3/7.  ^e^ The initial COD value was set close to the condition of mixture of sludge and manure at VS ratio 3/7 and pH 9.0. | | | | | |

**Table S2 Synergistic effect of main organic compounds in sewage sludge and cattle manure on VFAs production at a fermentation time of 12 d ^a^**.

| Model substrates | VFAs concentration |
| --- | --- |
| B ^b^ | 4.96 ± 0.25 |
| B+C+H+L ^c^ | 2.88 ± 0.15 |
| B+C+H+L ^d^ (VS ratio=3/7) | 4.13 ± 0.17 |
| B+C+H+L ^e^ (VS ratio=3/7), pH=9 | 4.96 ± 0.25 |
| ^a^ The unit is g-COD/L, and the TS refers to the initial TS. The data are the averages and their standard deviations in duplicate tests. B: BSA; D: Dextran; C: Cellulose; H: Hemicellulose; L: Lignin.  ^b^ The initial COD value was set close to the condition of sole sludge.  ^c^ The initial COD value was set close to the condition of sole manure.  ^d^ The initial COD value was set close to the condition of mixture of sludge and manure at VS ratio 3/7.  ^e^ The initial COD value was set close to the condition of mixture of sludge and manure at VS ratio 3/7 and pH 9.0. | |

**Table S3. Sequence Diversity and Library Coverage Estimates.**

| **Sample** | **Reads** | **OTU** | **Ace** | **Chao** | **Coverage** | **Shannon** | **Simpson** |
| --- | --- | --- | --- | --- | --- | --- | --- |
| a | 8969 | 535 | 622 | 618 | 0.985952 | 3.59 | 0.1979 |
| b | 7147 | 333 | 406 | 401 | 0.987827 | 3.57 | 0.0963 |
| c | 11643 | 590 | 676 | 673 | 0.988749 | 4.47 | 0.0344 |
| d | 5172 | 32 | 102 | 58 | 0.997100 | 0.76 | 0.7101 |
| e | 5172 | 42 | 61 | 55 | 0.997680 | 0.84 | 0.7016 |
| f | 5172 | 27 | 34 | 34 | 0.998453 | 0.68 | 0.7453 |

**Fig. S1 - Effects of VS ratios on the acetic acid production during the 18-day fermentation time. Error bars represent standard deviations of triplicate tests.**

**Fig. S2 - Rarefaction curves of bacterial sequences (a) and archaea (b) sequences from the fermentation reactors of sludge (blue solid), manure (black solid) and mixture (red solid). The OTUs were defined by clustering sequences at the dissimilarity levels of 3%.**

**Fig. S3 - Shannon diversity curves of bacterial sequences (a) and archaea (b) sequences from the fermentation reactors of sludge (blue solid), manure (black solid) and mixture (red solid).** **The OTUs were defined by clustering sequences at the dissimilarity levels of 3%, respectively.**

**References**

1. Guo, X., Wang, C., Sun, F., Zhu, W. & Wu, W. A comparison of microbial characteristics between the thermophilic and mesophilic anaerobic digesters exposed to elevated food waste loadings. Bioresour. Technol. **152**, 420–428 (2014).

2. Zhang, T., Shao, M. & Ye, L. 454 pyrosequencing reveals bacterial diversity of activated sludge from 14 sewage treatment plants. *ISME J.* **6**, 1137–1147 (2012).

3. Pruesse, E. *et al.* SILVA: a comprehensive online resource for quality checked and aligned ribosomal RNA sequence data compatible with ARB. [*Nucleic Acids Res.*](http://www.medsci.cn/sci/submit.do?id=a0d95432) **35**, 7188–7196 (2007).

4. Liao, X. *et al.* Pyrosequencing analysis of bacterial communities in drinking water biofilters receiving influents of different types. Process Biochem. **48**, 703–707 (2013).

1.  [↑](#footnote-ref-2)
